# 

**Published:** 1799-04

**Authors:** 


### *List of Medical Promotions in the Army.*

——— Roberts, M. D. Physician to the Forces, to be Assistant Inspector of Hospitals in the Leeward Islands, in the room of ——— M'Intire, retired on half-pay.

Dr. Moore, to be Inspector of Hospitals in Portugal.

Dr. Robertson, Inspector of Hospitals in Portugal, is removed to the same office in Minorca.

Surgeon Henry Worth, of the Eirst Foot Guards, is appointed Surgeon to the Savoy prison.

Surgeon Wm. Holmes, of the 5th Foot, to be Surgeon to the forces in Lower Canada.

Mr. Thomas Thompson, to be Assistant Surgeon to the 21st Regiment of Light Dragoons, in the room of Mr. J. Woodcock, deceased.

Assistant Surgeon Thomas Nixen, to be Surgeon to the First Regiment of Foot Guards, *vice* Worth, appointed to the Savoy Prison.

Mr. Samuel Watson, to be Assistant Surgeon, *vice* Nixen.

Mr. Pooler, to be Assistant Surgeon to the 17th Regiment of Foot.

Mr. Francis, to be Surgeon to the 67th Reglment, *v.* Fergusson, promoted to the 5th Foot.

14th Dragoons. Assistant Surgeon Thomas Kidd, to be Assistant Surgeon *v.* ——— Newman, placed on half-pay.

2d Battalion Breadalbane Fencibles, Mr. D. M'Leod, late of the First Batt. to be Surgeon.

Mr. Lewis Leese, to be Surgeon to the Volunteer Association of the Ward of Bread street, London.

85th Foot. ——— Abercrombie, to be Assistant Surgeon, *v.* Blyth, resig.

Mr. R. Colmer, to be Surgeon to the North Petherten Volunteers.

5th Foot. Surgeon Wm. Fergusson, to be Surgeon, *v.* Holmes.

Mr. Wm. Lewis, to be Surgeon to the Sussex Fencible Cavalry, *v.* Thomas, resigned.

Mr. Henry Reed, to be Surgeon to the 89th Foot, *v.* Rouch, retired.

### A LIST OF NEW MEDICAL PUBLICATIONS.

Annals of Medicine for the year 1798: Exhibiting a concise View of the latest and most important Discoveries in Medicine and Medical Philosophy. By ANDREW DUNCAN, Sen. M. D. and ANDREW DUNCAN, Junr. M. D. Fellows of the Royal College of Physicians, Edinburgh. Vol. III. pp. 556. 8vo. *Edinburgh*, Mudie; *London*, Robinsons; *Hamburg*, Perthes. Price 7s.

Contributions to Physical and Medical Knowledge, principally from the West of England: collected by THOMAS BEDDOES, M. D. pp. 539. 8vo. *Bristol*, Cottle; *London*, Longman and Rees.

Lectures

Lectures on Diet and Regimen; being a systematic inquiry into the most rational Means of preserving Health, and prolonging Life, together with physiological and chemical Explanations, calculated chiefly for the use of Families, in order to banish the prevealing Abuses and Prejudices in Medicine. By A. F. M. WILLICH, M. D. &c. pp. 643. 8vo. *London*, Longman and Rees. (Price 9s. in Boards.)

Biographia Medica: or Historical and Critical Memoirs of the Lives and Writings of the most eminent medical Characters, that have existed from the earliest Account of Time, to the present Period; with a Catalogue of their literary Productions. By BENJAMIN HUTCHINSON, Member of the Medical Society of London, of the Physical Society of Guy's Hospital, and of the London Company of Surgeons. Two vols. 8vo. *London*, Johnson. Price 16s. in Boards.

A Defence of the Cæsarean Operation, with Observations on Embryulcia, and the Section of Symphysis Pubis; addressed to Mr. W. SIMMONS, of Manchester, author of Reflections on the Propriety of performing the Cæsarean Operation: containing some new Cases, and illustrated by seven Engravings. By JOHN HULL, M. D. &c. *Printed at Manchester*, 1799, and sold in *London* by Bickerstaff, and *Edinburgh*, by Mudie.

### FOREIGN PUBLICATIONS.

*Collection de nouveaux livres elementaires, &c.* A Collection of new elementary works: No 1. Containing the wonderful structure of the human body, or Elements of Anatomy, for the use of youth. By L. F. JAUFFRET. One volume 8vo. (1 Livre 80 centimes) *Paris*, Dugour and Durand.

*Des Maladies des Enfans*,—Of the Diseases peculiar to Children, Girls, Pregancy, and Child-birth. By N. CHAMBON, First Physician to the Army, &c. In four divisions; each two small volumes, 8vo. and sold separately, Price 10 livres. *Paris*, Dugour and Durand.

*Meteorologie des Cultivateurs*, &c. the Farmer's Meteorology, with an advice to the inhabitants of the country, relative to the preservation of health, and banishment of prejudice. Duodecimo, (1 livre) *Paris*. Fuchs.

*Physische Ketzereyen, &c.* Heresies in Natural philosophy; or an attempt to introduce a more simple and easy method of illustration in Physics; by J. G. G. RUDIGERN, &c. 8 vo. 128 pp. 1799. *Leipzig*. Weygand. (Pr. 8 gr.

*Journal der Pharmacie*, &c. The Pharmaceutical Journal, for the use of Physicians, Apothecaries, and Chemists: by Dr. J. B. TROMMSDORFF. Volume fifth. No. I. and II. together 610 pp. 8vo. 1799. *Leipzig*. Crusius. (1 Rixdoll. 16 gr. or about 6s.)

*** *Want of room again obliges us to postpone the Account of several valuable new Publications, to a future Number.*

